# Bendable Electro-chemical Lactate Sensor Printed with Silver Nano-particles

**DOI:** 10.1038/srep30565

**Published:** 2016-07-28

**Authors:** Md Abu Abrar, Yue Dong, Paul Kyuheon Lee, Woo Soo Kim

**Affiliations:** 1 Stretchable Device Laboratory, School of Mechatronic Systems Engineering, Simon Fraser University, BC, Canada; 2 NTS Research & Inc., Coquitlam, BC, Canada

## Abstract

Here we report a flexible amperometric lactate biosensor using silver nanoparticle based conductive electrode. Mechanically bendable cross-serpentine-shaped silver electrode is generated on flexible substrate for the mechanical durability such as bending. The biosensor is designed and fabricated by modifying silver electrode with lactate oxidase immobilized by bovine serum albumin. The in-sensor pseudo Ag/AgCl reference electrode is fabricated by chloridization of silver electrode, which evinced its long-term potential stability against a standard commercial Ag/AgCl reference electrode. The amperometric response of the sensor shows linear dependence with lactate concentration of 1~25 mM/L. Anionic selectivity is achieved by using drop-casted Nafion coated on silver electrode against anionic interferences such as ascorbate. This non-invasive electrochemical lactate sensor also demonstrates excellent resiliency against mechanical deformation and temperature fluctuation which leads the possibility of using it on human epidermis for continuous measurement of lactate from sweat. Near field communication based wireless data transmission is demonstrated to reflect a practical approach of the sensor to measure lactate concentration portably using human perspiration.

Lactate is one of the cardinal metabolites produced during anaerobic phase of glycolysis. Under anaerobic conditions pyruvate converts to lactic acid by lactate dehydrogenase^1^. Lactic acid later gets dissociated into lactate at physiologic pH ranges. Lactate plays significant roles in clinical medicine and sport physiology^2^,^3^, neuron-glia metabolic interaction^4^, fermentation processes^5^, food and wine industry^6^. Decreased tissue oxygenation and lactic acidosis could cause ventricular failure and drug toxicity^7^. Breaking down of glycogen feeds the body necessary energy in the form of Adenosine triphosphate (ATP) in a process commonly known as glycolysis. With sufficient oxygen supply, ATP is produced constantly from pyruvate according to Krebs cycle^8^. During intense physical exercise aerobic metabolism cannot provide the energy needed for the human body all by itself, as oxygen supply becomes insufficient. That’s when anaerobic process kicks in and pyruvate coverts to lactic acid by lactate dehydrogenase and produces plethora of energy^9^. This process is also referred as ‘lactic acidosis’, causing an increase level of lactate in blood^10^. Lactate concentration in a healthy person may vary from 0.6 mM to 2 mM under resting condition^11^, but intense physical exercise could bump it up to as high as 10 mM^12^. Blood lactate can also increase in anomalous pathogenic patients, including the patients suffering from cardiac diseases and diabetes^11^. In cancer cells, rate of glycolysis increases even under aerobic condition which is commonly known as Warburg Effect^13^. This would increase the lactic acid level in the body, leading to acidosis. Researchers have hypothesized that this may have some effects on tumor proliferation and invasion, providing tumor cell competitive advantages^14^. Testing the lactate level can determine whether a person’s acid-base (pH) balance has been disrupted due to increase in lactate level. There are several diseases that could cause lactic acidosis such as cancer, sepsis, diabetes, and cardiovascular diseases. It is thus of utmost importance to monitor lactate concentration particularly in endurance-based physical activities and intensive care units.

Different measurement techniques and sensors have been developed for lactate quantification, such as colorimetric assays^15^, non-enzymatic method using HPLC^16^, integrated sensors in contact lenses^17^, or tattoos^18^ as noninvasive attempts. Commonly used sensors have been around for years which usually depend on blood draw using fingerpricks. Using blood as analyte lacks convenience, especially for continuous measurement during physical activities and also inherently possesses its drawback of invasiveness. Researchers have been exhorting on noninvasive techniques to measure lactate concentrations using bodily fluids such as tear, saliva, and sweat. A contact lens with an integrated Ti/Pd/Pt-based sensor has been demonstrated to measure lactate concentration using tear^17^. Another most common source of lactate is perspiration. Sweat lactate stems from eccrine gland metabolism, increase in exercise intensity accelerates increase in lactate concentration in sweat from ~10 mM to as high as ~25 mM^19^. Besides, there is strong relationship between lactate produced in blood and active muscle sweat^12^. Human sweat thus could successfully be employed as an excellent alternative analyte for noninvasive and continuous lactate measurement^18^. An electrochemical tattoo biosensor has been recently developed using carbon fiber and carbon nanotubes for continuous lactate measurement utilizing sweat^18^ which shows good linearity in range 1–20 mM. This amperometric sensor is tested under bending load and demonstrates high reliability. Table 1 presents similar studies among different noninvasive amperometric lactate sensors using immobilized lactate oxidase. It can be noted that the present work is intended to the fabrication of all three electrodes using one material with direct stamping of AgNPs. Also, the linear range of the sensor is observed relatively broader than a lot of other nonivasive amperometric lactate sensors.

Lactate oxidase (LOD) is most commonly used as an enzyme, which breaks down lactate into pyruvate and hydrogen peroxide. Hydrogen peroxide later oxidizes on electrode surface and produces amperometric current proportional to amount of lactate present in perspiration^20^. Usually relatively higher potential is needed for LOD-based analytical detection of hydrogen peroxide and overpotential is quite salubrious for interferences from other electroactive species in real sample^18^. Moreover conventional amperometric detection is also limited by slow kinetics of electrodes^21^.

High surface-to-volume ratio of nano-materials ushers in a new era for biosensor research to overcome the limitations mentioned above. Several researches have successfully utilized nano-materials and corroborated the advantages of using them over conventional materials in electrode fabrication and modification such as nitrogen doped carbon nanotubes (CNT)^22^, MnO_2_-modified vertically aligned multi-walled carbon nanotubes^23^, CNT/Nafion modified glassy-carbon electrodes^24^, Pt nanoparticle-loaded carbon nanofiber^25^, Au and Pt nanoparticles^26^. Silver nanoparticles (AgNPs) also attracted enormous interest for biosensor fabrication^27^. A glucose biosensor is demonstrated using AgNP to modify platinum electrode, which shows significant increase in current response^27^. Hydrogen peroxide sensor using glassy carbon electrodes (GCE) modified with electrodeposited AgNPs shows enhanced sensitivity and stability^28^. AgNPs show potential to increase activity of immobilized enzyme as well^29^.

One of the major problems with using lactate oxidase (LOD) is its limited stability. LOD tends to lose its activity quickly when it is removed from its natural matrix^30^. Therefore, successful immobilization of the enzyme is a vital step to fabricate biosensor with high performance. Several immobilization techniques have been developed to immobilize enzymes over past years^31^,^32^,^33^. Bovine serum albumin (BSA) is serum albumin globular protein which is well known for its properties to immobilize LOD with the help of crosslinking agent glutaraldehyde (GA)^34^. Nafion is sulfonated fluropolymer-copolymer incorporating hydrophobic tetrafluoroethylene (Teflon) backbone terminated with sulfonate acid group side chains^35^. Because of the existing negatively charged group, Nafion membrane can be used to exclude anionic interfering species such as ascorbate^36^.

In this work, we demonstrate lactate monitoring with a AgNP-based simple amperometric flexible sensor modified with BSA/GA to immobilize LOD. Nafion membrane is integrated for interference study. When it comes to using a sensor on human body (e.g. on skin) for continuous monitoring, the sensor must be able to conform to the continuous mechanical deformation. We utilize cross-serpentine-shaped pattern of AgNP electrodes for greater flexibility. Cross-serpentine-shaped electrodes with lower moment of inertia have been proven to be more stretchable and bendable. They also improve contact to the skin with much lower impedance^37^. Experiment for sensor characteristics related to temperature variation is also performed. Near Field Communication (NFC)-based signal transduction, conditioning and processing unit is demonstrated for successful and reliable wireless transmission of the sweat sensor measurement data.

## Results and Discussion

### AgNP-Electrodes, Shape and Flexibility

**Figure 1:**
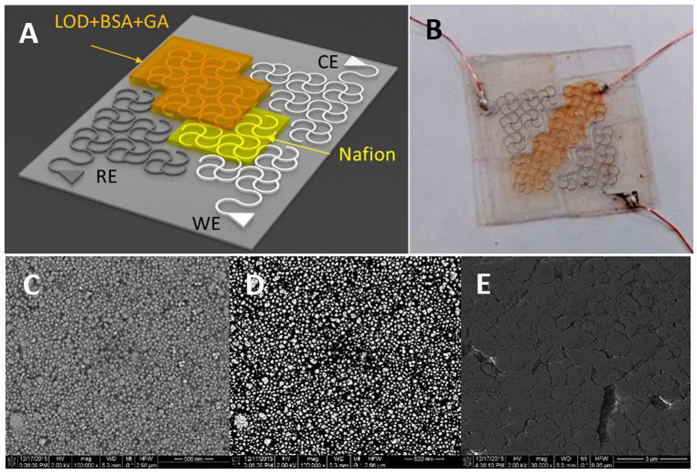
Images of fabricated and modified electrodes. (**A**) Schematic illustration of bendable electro-chemical sensor for monitoring sweat lactate, (**B**) Printed sweat lactate sensor (**C**) Surface morphology of working electrode (**D**) B/W modified picture of (**C**), **(E)** Reference electrode surface with Chloridized AgNPs for 5 min.

AgNPs are well known for their excellent electrochemical properties and catalytic activity. Electrochemical sensors incorporating AgNPs are demonstrated to be able to provide higher response with low response time and lower detection limit. AgNPs electrodeposited gold interdigital electrodes show excellent promises of higher sensitivity towards hydrogen peroxide^38^. Electrodes fabricated from metal nanoparticles exhibit larger surface-to-volume ratio compared to the ones fabricated with their bulk metal counterpart which means presence of more potential sites for reaction to take place. Figure 1(B) shows the surface roughness of cured AgNPs transferred onto the flexible substrate. Root mean square (rms) value for surface roughness of AgNP electrode is found to be 313.7 nm which is quite higher than rms roughness of the modified Ag/AgCl reference electrode (164.018 nm for 5 min long modified electrode).

Printed AgNPs’ densified character due to direct stamping technique plays a significant role in increasing the conductivity of electrodes. We employed simple spraying of AgNPs followed by stamping and thermal annealing for electrode fabrication^39^. According to classical power law, conductivity of composite material could be expressed by the following equation^40^.


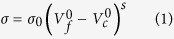


where *σ*_*0*_ is conductivity of filler, *V*_*f*_^*0*^ is volumetric fraction of filler, *V*_*C*_^*0*^ volumetric fraction of percolation threshold and *s* is critical exponent. As *Vc*^*0*^is constant, the conductivity depends on volumetric filler, which is AgNPs in this study. Direct stamping can greatly increase this property of electrode, which eventually helps to increase conductivity. AgNPs densification during the stamping also bears upon the mechanical property of the electrodes. Electrodes fabricated by direct stamping process exhibit superior behavior to the ones fabricated by inkjet printing in terms of greater flexibility and higher density. The problem inkjet print often encounters is the coffee ring effect, because of low ink concentration, which consequently leads to electrodes with irregular thickness and weak mechanical properties^41^. No physical pressure is applied in inkjet printing, ergo nanoparticles would get less of a chance to be packed tightly to form a continuous highly conductive pathway. In contrast, direct stamping shows superior characteristics confronting the downsides of inkjet printing. Electrodes fabricated by direct stamping were subjected to stretching up to 8% strain without decreasing electrical performance. Crack initiation was not observed up until 8% strain and it maintained excellent conductivity hitherto, which is much higher than other conventional printing methods, such as inkjet and screen printing^42^. Applied pressure from stamping makes adjacent AgNPs come closer to each other and become densely packed, hence lessen the presence of pores in the structure, which essentially means less availability of sites for crack initiation.

All three electrodes (working, counter and reference) are fabricated in cross-serpentine pattern for achieving high flexibility and conformal contact on the human epidermis by means of continuous measurement. Electrodes with this pattern are demonstrated to be much stretchable to their flat solid counterparts. Bending stiffness of a beam is available to be estimated using following equation^43^:


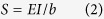


where *E* is elastic modulus, *I* is moment of inertia of bending cross-section and *b* is width of the film. Moment of inertial is proportional to cross-sectional area and serpentine shaped film has smaller cross sectional area than straight flat film which leads to less stiffness i.e. better bendability in serpentine shaped films^37^. Electrodes with cross-serpentine pattern are able to develop higher strain than flat electrodes, when same loading is applied. As loading increases, the strain observed in cross-serpentine pattern rises with it as opposed to straight flat’s almost unaltered strain. Upon stretching, cross-serpentine electrodes tend to open up in both directions but flat electrodes stay still. This behavior of cross-serpentine shaped thin electrodes insinuates their prospect to be successfully used on human epidermis due to very little relative movement expected from their conformity. Another important headway of cross-serpentine shaped conformal electrodes is generation of lower impedance on account of top-drawer contact between skin and electrode^37^.

### Optimization of Reference Electrode

A reference electrode for amperometry needs to show long-term stability, which is characterized by potential stability over long period of time. Commercial Ag/AgCl reference electrodes with saturated KCl are known to be adept in this regard. But such reference electrodes containing a solution makes it quixotic to be used in biosensors. Pseudo reference electrode is fabricated simply by modifying AgNP based electrodes with Clorox^®^ bleach (8.25%). Three different samples were prepared by dipping them for 1 min, 5 min and 10 min in bleach. Figure 1(C,E) are scanning electron microscopy (SEM) images of unchloridized and chloridized AgNPs (5 min). Average diameter of synthesized AgNPs is around 50 nm. Upon chloridization, AgCl particles start to form on AgNP surfaces and as the chloridization time was increased, AgCl tends to enshroud AgNPs springing much smoother surface morphology. Table 2 provides the percentage amount of silver and chlorine in different chloridized samples achieved from Energy-dispersive X-ray spectroscopy (EDS).

**Figure 2:**
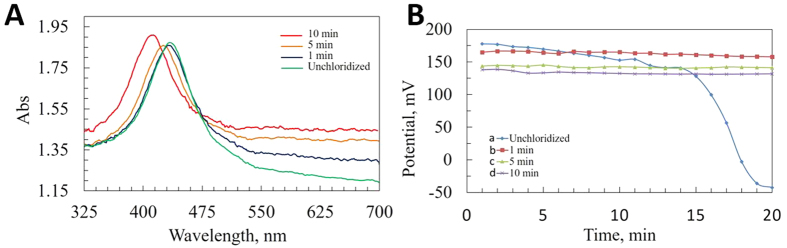
Reference electrode characterization and stability test. (**A**) UV-Vis spectra of chloridized AgNPs. Resonance peak for unchloridized AgNP at 440 nm. Shifted absorption at shorter wavelength than 440 nm in chloridized AgNP samples, (**B**) Potential stability test of different chloridized samples with saturated KCl. Unchloridized AgNPs shows erratic behavior in potential stability.

Scattering and absorbing light in visible range is one of the characteristics of noble metal nanoparticles^44^. Nanoparticles’ distinctive color depends on their morphology and size unlike dyes and pigments. When the conductive electrons of nanoparticles are excited by an external light at specific frequency, they undergo a collective oscillation which is known as surface plasmon resonance^45^. Due to this oscillatory phenomenon, nanoparticles show strong absorption and scattering behavior. Optical and physical properties of noble metal, nanoparticles are significantly different from their bulk metal counterparts^46^,^47^. Silver nanoparticles show phenomenal efficacy towards absorbing and scattering light in visible range^48^. Ultraviolet–visible spectroscopy (UV-Vis) is performed to characterize different chloridized samples. Spectral response of silver nanoparticles as a function of chloridization time is presented in Fig. 2(A). Silver nanoparticles show a resonance peak at around 440 nm which corresponds to their specific shape and size. Ag/AgCl shows stronger absorption in the visible range and as the chloridization time increases their absorption trait gets stronger. The peak also tends to shift to shorter wavelengths with the increase of chloridization time.

Long-term stability performance tests are performed in saturated KCl using a BASi^®^ standard Ag/AgCl reference electrode as counter electrode. All the electrodes are tested three times each in a span of 20 mins. Unchloridized electrode with unmodified AgNPs shows mercurial behavior in its potential stability (Fig. 2(B)). Electrical potential changes significantly at every minute and after 14 min it plummets down from 140.8 mV to −42.4 mV over just 5 min of duration. Modified Ag/AgCl electrodes show excellent performance keeping their potential constant with insignificant variations. Although the sample (c) comes out to be topnotch in the first experiment, sample (b) vanquishes others when it comes to three experiments all together. Standard deviations for samples (a), (b), (c) and (d) are calculated to be 108.05, 7.81, 12.95 and 12.57. Modified AgNP electrode sample (b) dipped in bleach for 1 min is thus used as reference electrode for our biosensor.

### *In Vitro* Evaluation of Lactate Sensor

The operating potential of the lactate sensor is selected using cyclic voltammetry (CHI1205B) technique with a scan rate of 50 mV/s between −1 to 1 V for 10 mM of lactate solution. All the lactate solutions are prepared in PBS (pH = 7.0). Amperometric studies are performed after 1 min incubation in the solution with step potential of 0.65 V. The oxidation peak is found at 0.65 V and is thus used as the operating potential for the sensor for all the subsequent studies. *In vitro* experiments were carried out by dropcasting and bearing off 200 μL of solution with different concentrations on the sensor each time.

**Figure 3:**
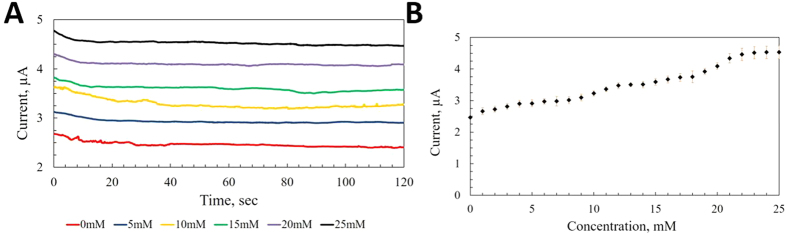
Amperometric responses of AgNP-based lactate sensor at 0.65 V vs Ag/AgCl reference electrode. (**A**) Current response vs lactate concentration (**B**) Calibration curve for responses at different lactate concentrations taken at 60^th^ s.

Figure 3(A) shows amperometric response of the biosensor upon addition of lactate with different concentrations. Current response rises as the concentration increases from 0 mM to 25 mM. Sensor response reaches to 90% of its final steady value within 50 seconds (response time). The response observed upon addition of 0 mM of lactate could be attributed as constant background current, I_0_ and can be corrected^49^. Sensor responses at 60^th^ seconds are plotted in Fig. 3(B) for different lactate concentrations. The calibration curve demonstrates linear relation with lactate concentration with average sensitivity of 256 nA mM^−1^ cm^−2^ and coefficient of determination of 0.9732. Linear response range of this AgNP-based lactate sensor is found much higher than a lactate sensor with multiwall carbon nanotube (MWCNT) and ZnO modified electrode (0.2–2 mM)^50^.

### Interference study, temperature dependence and bending test

**Figure 4:**
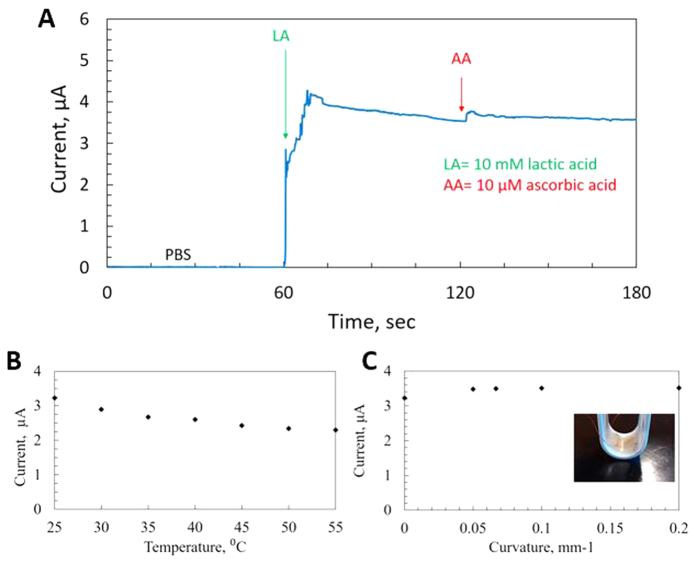
Selectivity and stability test of sensor (0.65 V vs Ag/AgCl pseudo reference electrode). (**A**) Interference test performed with 10 μM of ascorbate at 120 s (**B**) Temperature dependence of the enzyme based sensor (**C**) Amperometric response of the sensor vs bending curvature (Inset: sensor under bending with 0.1 mm^−1^).

Nafion- a sulfonated fluoropolymer has a hydrophobic backbone of polytetrafluoroethylene (PTFE) with side chains containing hydrophilic sulfonic acid groups^51^. The hydrophilic sulfonic acid group –SO_3_H dissociates in the presence of water and the proton (H^+^) bonds with water molecule creating hydromium ion (H_3_O^+^) leaving nafion as negatively charged^52^. Nafion as a negatively charged polymer, is commonly known to be applied to electrochemical biosensors to block anionic interferences and increase selectivity^11^,^53^,^54^. Fabricated AgNP working electrode is coated with nafion and interference test with ascorbate is performed. Figure 4(A) shows the amperometric responses of the sensor to ascorbate. The sensor does not show any current response when PBS is added. As soon as 10 mM of lactate is added, an increase of current response is noticed while contribution from mean physiological level of interference (10 μM of ascorbate^55^) is negligible. The excellent anti-interference performance of the sensor stems from utilized Nafion and lower working potential requirement.

Enzymes’ activity strongly depends on the ambient condition, where they are being used and they are often susceptible to thermal denaturation. However, immobilized enzymes tend to show different thermal characteristics than when they are in their free state in nature^56^. Thermal stability of the developed lactate sensor is investigated at elevated temperatures. Figure 4(B) presents the current response from same lactate level (10 mM) at different temperatures (25 °C to 55 °C). Although significant drop in response is observed between 25 °C and 55 °C (~28.86%), the drop of response in the physiological temperature range between 35 °C and 40 °C is minimal (~2.84%).

Wearable conformal sensors on epidermis are expected to be experiencing regular mechanical deformation due to movements of human body. Hence these sensors constantly need to prove their robustness against these disfigurations. AgNP-based stamped electrodes could be stretched up to 20% strain with insignificant change in electrical performance^37^. The developed lactate sensor is subjected to mechanical bending with different curvature values. Figure 4(C) represents the sensor’s behavior in response to bending curvatures. The maximum deviation was noticed to be only 9.08% at 0.2 mm^−1^. This excellent bendability of the sensor is the result of the cross-serpentine structure with relatively smaller cross-sectional area of the fabricated electrodes. The sensor’s electrical performance has been tested in cyclic bending. And the resistance change of sensors has been noticed as only 2.55% at 0.1 mm^−1^ of curvature with inward bending and 3.55% with outward bending. A repeatedly bendable directly stamped silver electrode has been reported^57^. The fabricated electrodes have been bent repeatedly to 8 mm of bending radius at 1 Hz for 10,000 cycles, which generates no significant change in electrical performance. The excellent flexibility of the silver electrode could be attributed to the densified packing behavior of direct stamping process with AgNPs.

When it comes to reliability of continuous measurement, wearable sensors should be able to reflect the analyte concentration over long period of time. However the stacks of different functional materials could be subjected to periodic decay as analyte solution is continuously added on to the sensor surface. This can be improved by applying an outer protective layer to keep the enzyme solution and anionic polymer from leaching out. Further modification of the sensor with biocompatible outer layer (e.g. chitosan) would not only improve the reusability of the sensor but also block other interferences and control diffusion of lactate flux.

### Electrochemical Signal Processing and Wireless Transmission

**Figure 5:**
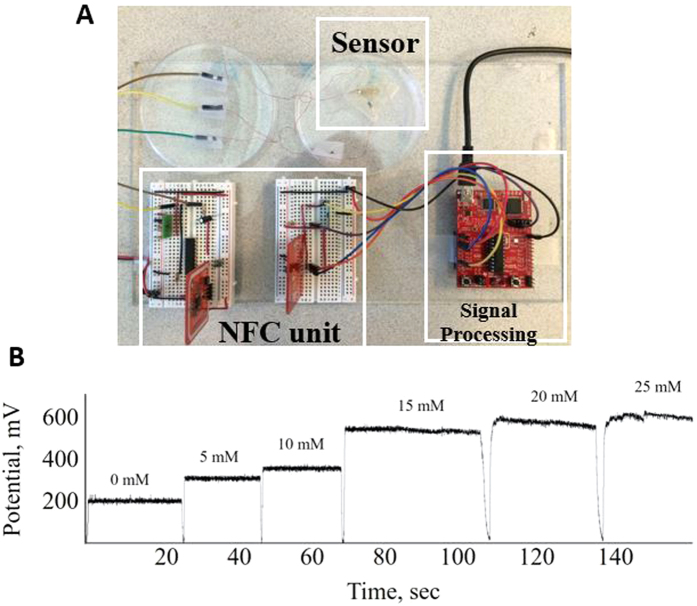
Image of sensor system and its data. (**A**) Photograph of the bendable sensor connected with signal processing unit and NFC transceiver module (**B**) Voltage Response of the sensor performance transferred to computer by NFC wireless transmission.

The electrochemical sensor output is in the form of current signal with an order of 10^−6^ (μA). In the respective current signal-conditioning path, a transimpedance amplifier to convert the signal current into voltage is used. Transimpendace amplifier (OPA380) has a low input impedance and a high output impedance. With a 660 KΩ feedback resistor, the converted voltage signal is amplified and has range from 0 to 3.3 V, while staying within input voltage range of analog-to-digit converter (ADC) inside microcontroller. A16-bit microcontroller (MSP430g2553) with 10-bit internal ADC is used. Therefore, the current sensing path is capable of measuring current levels as low as 4 nA. Conditioned voltage then goes into internal ADC, gets converted from analog voltage to a digital data. Microcontroller collects data and sends a package to NFC module via UART port. Two PN532 NFC modules are used to exchange data wirelessly. NFC transmitter wirelessly transmits the data package with encoded ADC values to NFC receiver at 2 Hz. Another MSP430 microcontroller is connected to NFC receiver to decipher the values. The received data package is finally decoded by it, and digital data is acquired on PC via serial communication interface with the help of MATLAB. Figure 5(B) represents the response of the sensor acquired from NFC module after testing with lactate solutions with different concentrations. It shows distinctive rise of almost steady response according to the increase in lactate concentration and sensor recovery time recorded was less than 2 seconds. Although the original sensor’s response from commercial potentiostat is quite linear similar to Fig. 3b, the corresponding wirelessly transmitted NFC voltage shows a bump around 15 mM point because we do not add bias protection for the transimpedence amplifier circuits. It is suspected that the output voltage from the wireless transmission in Fig. 5 is boosted a bit when the current from the sensor reaches a certain level. This issue may be resolved in future prototypes of wireless lactate sensor.

## Materials and Methods

### Reagents

Silver acetate, phenylhydrazine, toluene (anhydrous, 99.8%), hexadecylamine (technical grade, 90%), Isopropanol (IPA), Nafion, Ethanol (anhydrous, ≤0.003% water), Lactate Oxidase from Pediococcus sp. (lyophilized powder, ≥20 units/mg solid), L-Lactic Acid, Bovine Serum Albumin (BSA) (pH 7, ≥98%), Glutaraldehyde (GA) (25% in H_2_O) were purchased from Sigma-Aldrich. Polyurethane (PU) was modified with Propylene glycol monomethyl ether acetate (PGMEA) (Sigma-Aldrich) and Photo initiator (Ciba DAROCUR 1173). Methanol (absolute, low acetone) was obtained from Alfa Aesar. Phosphate buffer solution (PBS, 0.1 M) was prepared with 0.1 M NaH_2_PO_4_ and 0.1 M Na_2_HPO_4_ in DI water.

### Synthesis of silver nanoparticles

Silver nanoparticles were synthesized as described elsewhere^39^ with a tinge of modification. Silver nanoparticles were synthesized by reducing silver acetate with phenylhydrazine. 28 gm of hexadecylamine; as a capping agent was slowly added into a flask with 32 ml of toluene at 65 °C. Then 4 gm of silver acetate and another portion of 28 gm hexadecylamine were dissolved in the solution. The solution was kept stirring and temperature was brought down to 60 °C over a period of 30 min. A solution of 1.73 gm toluene and 1.4 gm of phenylhydrazine was dripped into the flask over 10 min. After 15 min, the flask was cooled down to 50 °C over 30 min. The prepared nanoparticle solution was washed using 100 ml isopropanol and 100 ml methanol. The washed solution was filtered and dried in vacuum oven at 60 °C overnight. Dried silver nanoparticles were scraped off and used for electrode fabrication.

### Electrode preparation

The AgNP electrodes were fabricated using direct stamping in conjunction with simple spray coating technique. Silicon master pattern with cross-serpentine pattern was prepared by conventional photolithography using SU-8 negative photoresist. This master pattern was used to replicate flexible PDMS stamps with the patterns as trenches. AgNP ink (3 wt%) was prepared using toluene as solvent and sprayed on the stamps using IWATA HP-CR air brush in a fume hood. Then, the adhesive tape was used to remove unwanted NPs from the protrusive areas of the stamp. A thin layer of UV-curable PU prepolymer (urethane acrylate 90 wt% and photo-initiator 5 wt% in PGMEA) was homogenously daubed on the stamps with AgNPs left in the trenches. Then the stamps were brought in contact with PET substrates and moderate pressure was applied. The entire assembly comprised of stamp, PU and PET was exposed to UV light for 7 mins. After that the substrates were peeled off the stamp and thus AgNPs get transferred with crosslinked hardened PU. They were then annealed at 160 °C for 1.5 hours. Surface area of the working electrode was measured as 32 mm^2^. Electrical connections were made using silver conductive epoxy and 0.19 mm enameled copper wires.

The next steps were to modify the working electrode with different functional layers. Nafion was received as a 20 wt.% mixture of lower aliphatic alcohols and water. It was dissolved in ethanol to prepare diluted (5% v/v) Nafion solution. To prepare the Nafion selective membrane, 20 μL of the solution was drop casted on the electrode surface and left it to dry for an hour. For the enzymatic matrix, 50 U of LOD was dissolved in 500 μL of PBS. The whole solution was equally separated into 25 aliquots; each containing 2 U of LOD in 20 μL solution and stored at −20 °C. Bovine serum albumin (BSA) was received as lyophilized powder. A total mass of 6 mg of BSA was dissolved in 40 μL of PBS and stored at 4 °C. Prepared 40 μL of BSA solution was mixed with 20 μL of LOD solution and stored at 4 °C. Glutaraldehyde (GA) was diluted into 10% solution with DI water. To have the enzymatic matrix, a 30 μL of GA was mixed with LOD/BSA concoction and 20 μL of LOD/BSA/GA solution was drop casted on Nafion coated electrode surface. After waiting for 2 hours, the prepared sensor was gently rinsed with PBS to get rid of excess GA.

Ag/AgCl pseudo reference electrodes were prepared by dunking AgNP-electrodes in 8.25% Clorox bleach for 1, 5 and 10 mins and then they were washed with DI water. Unmodified, unchloridized cross-serpentine-patterned conductive AgNP-electrode was used as counter electrode.

### Characterization Methods of Printed Ag Electrode

All electrochemical experiments were performed on a CHI 1205B electrochemical analyzer (CH Instruments Inc.). The electro-chemical sensor works in a conventional three electrode system. All the three electrodes were fabricated with silver nanoparticles using IWATA HP-CR air brush as noted before. Working electrode was modified with enzymatic matrix. And AgCl in the reference electrode was prepared with bleach. Scanning Electron Microscopy (SEM) images were obtained using Nova NanoSEM 430 SEM which was also used for energy dispersive spectroscopy (EDS). Varian Cary^®^ 50 UV-Vis Spectrophotometer was used for UV-visible spectroscopy (UV-Vis).

## Additional Information

**How to cite this article**: Abrar, M. A. *et al*. Bendable Electro-chemical Lactate Sensor Printed with Silver Nano-particles. *Sci. Rep.* **6**, 30565; doi: 10.1038/srep30565 (2016).

## Figures and Tables

**Table 1:** Noninvasive amperometric lactate sensors.

Electrode Materials	Sensing Fluid	Sensor Type	Sensitivity	Fabrication Method	Linear range	Interference study	Ref.
Silver Nanoparticles	Sweat	Flexible and bendable	262 nA mM^−1^ cm^−2^	Direct stamping	1–25 mM	Yes	Current research
Carbon nanotubes and Ag/AgCl	Sweat	Flexible and bendable	644.2 nA/mM	Screen printing	1–20 mM	Yes	18
Graphite with addition of Prussian Blue and Ag/AgCl	Saliva	Printable on curved surface	553 nA/mM	Screen printing	0.1–1 mM	Yes	58
Ti/Pd/Pt integrated on contact lens	Tear	Rigid film type	53 μA mM^−1^ cm^−2^	E-beam evaporation	0–1 mM	Yes	17

**Table 2:** Chemical characterization of different chloridized AgNP samples.

Chloridization time	Ag wt%	Cl wt%
0 min	85.99	0
1 min	88.31	3.87
5 min	79.60	8.82
10 min	73.79	10.26
